# Procalcitonin use in febrile children attending European emergency departments: a prospective multicenter study

**DOI:** 10.1186/s12887-025-05483-1

**Published:** 2025-03-01

**Authors:** Dorine M. Borensztajn, Joany M. Zachariasse, Enitan D. Carrol, Ruud G. Nijman, Ulrich von Both, Marieke Emonts, Jethro Herberg, Benno Kohlmaier, Michael Levin, Emma Lim, Ian K. Maconochie, Federico Martinón-Torres, Marko Pokorn, Irene Rivero-Calle, Aleksandra Rudzāte, Chantal D. Tan, Maria Tsolia, Clementien Vermont, Dace Zavadska, Werner Zenz, Henriette A. Moll, On behalf of PERFORM consortium: Personalised Risk assessment in febrile children to optimise Real-life Management across the European Union, Dorine M. Borensztajn, Joany M. Zachariasse, Enitan D. Carrol, Ruud G. Nijman, Ulrich von Both, Marieke Emonts, Jethro Herberg, Benno Kohlmaier, Michael Levin, Emma Lim, Ian K. Maconochie, Federico Martinón-Torres, Marko Pokorn, Irene Rivero-Calle, Chantal D. Tan, Maria Tsolia, Clementien Vermont, Dace Zavadska, Henriette A. Moll, Nienke N. Hagedoorn, W. Dik, Stéphane Paulus, Elizabeth Cocklin, Rebecca Jennings, Joanne Johnston, Simon Leigh, Karen Newall, Sam Romaine, Aubrey Cunnington, Tisham De, Myrsini Kaforou, Victoria Wright, Lucas Baumard, Evangelos Bellos, Giselle D’Souza, Rachel Galassini, Dominic Habgood-Coote, Shea Hamilton, Clive Hoggart, Sara Hourmat, Heather Jackson, Stephanie Menikou, Naomi Lin, Samuel Nichols, Ivonne Pena Paz, Priyen Shah, Ching-Fen Shen, Ortensia Vito, Clare Wilson, Amina Abdulla, Ladan Ali, Sarah Darnell, Rikke Jorgensen, Sobia Mustafa, Salina Persand, Laura Kolberg, Manuela Zwerenz, Judith Buschbeck, Christoph Bidlingmaier, Vera Binder, Katharina Danhauser, Nikolaus Haas, Matthias Griese, Tobias Feuchtinger, Julia Keil, Matthias Kappler, Eberhard Lurz, Georg Muench, Karl Reiter, Carola Schoen, Lucille Valentine, Karen Allen, Kathryn Bell, Adora Chan, Stephen Crulley, Kirsty Devine, Daniel Fabian, Sharon King, Paul McAlinden, Sam McDonald, Anne McDonnell, Ailsa Pickering, Evelyn Thomson, Amanda Wood, Diane Wallia, Phil Woodsford, Frances Baxter, Ashley Bell, Mathew Rhodes, Rachel Agbeko, Christine Mackerness, Bryan Baas, Lieke Kloosterhuis, Wilma Oosthoek, Tasnim Arif, Joshua Bennet, Kalvin Collings, Ilona van der Giessen, Alex Martin, Aqeela Rashid, Emily Rowlands, Gabriella de Vries, Fabian van der Velden, Lucille Valentine, Mike Martin, Ravi Mistry, Mistry Zenz, Nina A. Schweintzger, Manfred G. Sagmeister, Daniela S. Kohlfürst, Christoph Zurl, Alexander Binder, Susanne Hösele, Manuel Leitner, Lena Pölz, Glorija Rajic, Sebastian Bauchinger, Hinrich Baumgart, Martin Benesch, Astrid Ceolotto, Ernst Eber, Siegfried Gallistl, Gunther Gores, Harald Haidl, Almuthe Hauer, Christa Hude, Markus Keldorfer, Larissa Krenn, Heidemarie Pilch, Andreas Pfleger, Klaus Pfurtscheller, Gudrun Nordberg, Tobias Niedrist, Siegfried Rödl, Andrea Skrabl-Baumgartner, Matthias Sperl, Laura Stampfer, Volker Strenger, Holger Till, Andreas Trobisch, Sabine Löffler, Antonio Salas, Fernando Álvez González, Cristina Balo Farto, Ruth Barral-Arca, María Barreiro Castro, Xabier Bello, Mirian Ben García, Sandra Carnota, Miriam Cebey-López, María José Curras-Tuala, Carlos Durán Suárez, Luisa García Vicente, Alberto Gómez-Carballa, Jose Gómez Rial, Pilar Leboráns Iglesias, Nazareth Martinón-Torres, José María Martinón Sánchez, Belén Mosquera Pérez, Jacobo Pardo-Seco, Lidia Piñeiro Rodríguez, Sara Pischedda, Sara Rey Vázquez, Carmen Rodríguez-Tenreiro, Lorenzo Redondo-Collazo, Miguel Sadiki Ora, Sonia Serén Fernández, Cristina Serén Trasorras, Marisol Vilas Iglesias, Mojca Kolnik, Katarina Vincek, Tina Plankar Srovin, Natalija Bahovec, Petra Prunk, Veronika Osterman, Tanja Avramoska, Anda Balode, Arta Bārzdiņa, Dārta Deksne, Dace Gardovska, Dagne Grāvele, Ilze Grope, Anija Meiere, Ieva Nokalna, Jana Pavāre, Zanda Pučuka, Katrīna Selecka, Aleksandra Sidorova, Dace Svile, Urzula Nora Urbāne, Maria Tambouratzi, Antonis Marmarinos, Marietta Xagorari, Kelly Syggelou, Ronald de Groot, Michiel van der Flier, Marien I. de Jonge, Koen van Aerde, Wynand Alkema, Bryan van den Broek, Jolein Gloerich, Alain J. van Gool, Stefanie Henriet, Martijn Huijnen, Ria Philipsen, Esther Willems

**Affiliations:** 1 Department of General Pediatrics, Erasmus MC-Sophia Children’s Hospital, Wytemaweg 80, Rotterdam, 3015 CN the Netherlands; 2 Department of Pediatrics, Noordwest Ziekenhuisgroep, Alkmaar, the Netherlands; 3 Department of Clinical Infection, Microbiology and Immunology, Institute of Veterinary and Ecological Sciences, University of Liverpool, Liverpool, UK; 4 Imperial College of Science, Technology and Medicine, Section of Pediatric Infectious Diseases, London, UK; 5 Dr. Von Hauner Children’s Hospital, Division of Pediatric Infectious Diseases, University Hospital, Ludwig-Maximilians-University (LMU), München, Germany; 6 Newcastle upon Tyne NHS Hospitals Foundation Trust, Great North Children’s Hospital, Paediatric Immunology, Infectious Diseases & Allergy; and Newcastle University, Translational and Clinical Research Institute, Newcastle Upon Tyne, UK; 7 Department of General Pediatrics, Medical University of Graz, Graz, Austria; 8 Hospital Clínico Universitario de Santiago de Compostela, Genetics, Vaccines, Infections and Pediatrics Research group (GENVIP), Santiago de Compostela, Spain; 9 Department of Infectious Diseases, University Medical Centre Ljubljana, Ljubljana, Slovenia; 10 Department of Pediatrics, Rīgas Stradiņa Universitāte, Children Clinical University Hospital, Riga, Latvia; 11 Second Department of Pediatrics, National and Kapodistrian University of Athens, P. And A. Kyriakou Children’S Hospital, Athens, Greece; 12 Department of Pediatric Infectious Diseases & Immunology, Erasmus MC-Sophia Children’s Hospital, Rotterdam, the Netherlands

**Keywords:** Procalcitonin, CRP, Biomarkers, Emergency Department

## Abstract

**Background:**

Studies on procalcitonin (PCT) for identifying sepsis were published as early as 1993 and since then, PCT has been the topic of over 8,500 studies. Several studies show PCT to be superior to CRP in differentiating invasive infections such as sepsis from viral infections, especially early in the disease course.

However, its actual use in clinical practice is poorly documented. Our aim was to study the use of PCT in febrile children attending the ED across Europe and compare this to the use of CRP.

**Methods:**

The MOFICHE/PERFORM study, a prospective multicenter study, took place at 12 European EDs in eight countries and included febrile children < 18 years. In this secondary analysis of nine participating EDs that used PCT, descriptive analyses were performed, describing the use of PCT in all febrile children and for different age groups, foci of fever and fever duration.

**Results:**

In total, 31,612 pediatric febrile episodes were available for analyses. Blood tests were performed in 15,812 (50.0%, range 9.6–92.6%)) febrile episodes. CRP was included in 98.3% of blood tests (range between hospitals 80–100%), while PCT was included in only 3.9% (range 0.1–86%).

PCT was most often performed in children below 3 months (12.0% versus 3.6% in older children, *p* < 0.001). PCT was used slightly more often in children with fever less than 24 h in comparison to children with a duration of fever ≥ 24 h (4.9% versus 3.4%, *p* < 0.001). Regarding clinical alarming signs, PCT was used most often in children with meningeal signs (7.0%) or a non-blanching rash (10.9%).

**Conclusion:**

Actual PCT use in febrile children at European EDs is limited and varies largely between hospitals. Possible explanations include lack of guidelines, limited availability, higher costs and lack of readiness to adapt new clinical strategies.

**Supplementary Information:**

The online version contains supplementary material available at 10.1186/s12887-025-05483-1.

## Introduction

Studies on procalcitonin (PCT) for identifying sepsis were published as early as 1993 and since then PCT has been the topic of over 8,500 studies, including systematic reviews showing its sensitivity and specificity are better than CRP in identifying severe bacterial infections in some groups of febrile children [1, 2]. In the literature on febrile children, the terms serious bacterial infections (SBI) and invasive bacterial infections (IBI) are frequently used to classify type of infections [2]. SBI is used to indicate a broad spectrum of conditions (e.g., bacterial meningitis, sepsis, bacteremia, urinary tract infections, pneumonia, bacterial gastroenteritis, bone or soft tissue infections), while IBI indicates the subgroup of most severe infections: bacterial meningitis, sepsis, and bacteremia [2].

Although procalcitonin (PCT) does not appear to provide added value over C-reactive protein (CRP) in identifying serious bacterial infections (SBI) in *all* febrile children [1, 3], several studies, including a systematic review published in 2024, demonstrate that PCT is superior to CRP in detecting invasive bacterial infections (IBI), such as sepsis, but not SBI, such as pneumonia, in febrile children without an apparent source [2, 4, 5]. This systematic review included 14 studies that separately analyzed IBI and SBI, defining IBI as the presence of a bacterial pathogen in blood or cerebrospinal fluid, while SBI encompassed a broader range of serious bacterial infections, varying across studies. This review concluded that PCT outperformed CRP in identifying IBI but not SBI [5].

This is especially the case early in the disease course [2, 4], due to the fact that concentration of PCT in the blood increases earlier than that of CRP [4]. Although most studies have focused on younger children (< 36 months old), at least one systematic review included children up to the age of 18 [2].

However, one of the drawbacks is that PCT is more expensive than CRP. Furthermore, PCT is not universally available at all EDs, possibly due to the fact that health services do not finance PCT due to the lack of official recommendations of its use and limited studies demonstrating a better cost-effectiveness ratio compared to CRP.

Actual PCT use at the pediatric ED is poorly documented and seems limited [6]. Our aim was to describe the actual use of PCT in febrile children attending the ED across Europe and compare this to CRP use.

## Methods

This study is a secondary analysis and is part of the MOFICHE study (Management and Outcome of Febrile children in Europe), which is embedded in the PERFORM study (Personalized Risk assessment in Febrile illness to Optimize Real-life Management across the European Union) [7]. MOFICHE is an observational multicenter study that evaluates the management and outcome of febrile children in Europe using routinely collected data [7].

### Patient and public involvement

Patients were not involved in the design of the study.

### Study population and setting

Twelve EDs from eight different countries participated in the MOFICHE study, nine of which use PCT routinely (Austria, Germany, Greece, Latvia, the Netherlands, Spain, Slovenia, and the United Kingdom (*n* = 2)). The three hospitals where PCT was not available were excluded. Participating hospitals were either tertiary university hospitals or large teaching hospitals [7]. Inclusion criteria were children aged 0–18 years presenting to the ED with fever (temperature > = 38.0° C) or a history of fever in the previous 72 h.

### Data collection and definitions

Data were collected for at least one year (January 2017-April 2018). Data collection ranged from one week per month to the entire month, depending on the number of ED visits per hospital [7]. Data were obtained from routine patient records and entered into an electronic case report form. Data included general patient characteristics (age, sex, comorbidity), laboratory tests performed (e.g. blood tests, urinalysis, rapid viral tests, cerebrospinal fluid sampling) and disposition (discharge, admission).

Focus of infection was allocated retrospectively by the local research teams based on patient records and was coded into predefined categories used by the previous MOFICHE/PERFORM studies as upper respiratory, lower respiratory, gastro-intestinal/surgical abdomen, urinary, skin/musculoskeletal, sepsis/meningitis, childhood exanthems/flu-like illness, inflammatory, undifferentiated fever or other. The presumed cause of the infection (e.g. viral, bacterial) was classified according to a predefined flowchart by the local research team [7].

### Data analysis

We performed descriptive analyses, comparing PCT and CRP use in all febrile children and in subgroups of different ages, different durations of fever and clinical alarming signs. Clinical alarming signs were defined as those alarming signs from the "red traffic light” symptoms for identifying risk of serious illness from the (NICE) guideline on fever that could be a symptom of an invasive bacterial infection (sepsis or meningitis): ill appearance, non-blanching rash, abnormal consciousness, meningeal signs or neurological signs [8].

Differences between groups in PCT use (with or without CRP) versus CRP use without PCT were assessed for children of different ages and for children with a different duration of fever using chi-squared-tests. Results were deemed significant with a *p*-value < 0.05.

## Results

In total, 31,612 pediatric febrile episodes were available for analyses (Table 1, appendix 1). Blood tests were performed in 15,812 (50.0%, range 9.6–92.6%) febrile episodes. CRP was included in 98.3% (*N* = 15,539) of blood tests, while PCT was included in only 3.9% (*n* = 620, range 0.1–86%, *p* < 0.001). In all subgroups regarding age or duration of fever, PCT was performed less often than CRP (Table 1, *p* < 0.001).

**Table 1:** Patient characteristics of children in which CRP and PCT were performed

	Number of episodes	Lab tests performed	CRP without PCT (%)	PCT with or without CRP (%)	*P*-value
**All children with fever: *N*=31,612**	31,612	15,812	14,959 (94.6)	620 (3.9)	
**Age**					*P* < 0.001
< 3 months	777	559	480 (85.9)	67 (12.1)	
3–36 months	15,740	7,848	7,485 (95.4)	285 (3.6)	
> 36 months	15,095	7,405	6,994 (94.4)	268 (3.6)	
**Comorbidity**					*P* < 0.001
No	27,036	13,047	12,535 (95.1)	482 (3.7)	
Yes	4,317	2,765	2,343 (84.7)	138 (5.0)	
Immunocompromised children	368	305	277 (90.8)	16 (5.2)	
**Duration of fever**					*P *< 0.001
< 24 h	10,426	4,780	4,469 (93.5)	236 (4.9)	
≥ 24 h	18,913	10,230	9,762 (95.4)	347 (3.4)	
**Triage category**					*P* < 0.001
Low urgent	22,902	9,905	9,332 (94.2)	409 (4.1)	
Intermediate urgent	6,970	4,564	4,320 (94.7)	194 (4.3)	
High urgent	1,740	1,343	1,307 (97.3)	17 (1.3)	
**Abnormal vital signs**					
Tachycardia (APLS reference ranges)	7,570	4,033	3,806 (94.4)	172 (4.3)	*P* = 0.193
Tachypnoea (APLS reference ranges)	5,526	2,724	2,537 (93.1)	154 (5.6)	*P* < 0.001
Low oxygen saturation ≤ 94%	728	434	412 (94.9)	19 (4.4)	*P* = 0.619
Capillary refill time ≥ 3 s	543	489	467 (95.5)	16 (3.3)	*P* = 0.453
**Clinical alarming signs**					
Ill appearance	5,654	4,621	4,458 (78.8)	120 (2.6)	*P* < 0.001
Non-blanching rash	1,034	607	524 (86.5)	66 (10.9)	*P* < 0.001
Abnormal consciousness	156	118	109 (92.4)	8 (6.8)	*P* = 0.108
Meningeal signs	128	114	105 (92.1)	8 (7.0)	*P* < 0.087
Neurological signs	126	104	102 (98.1)	1 (1.0)	*P* = 0.713
**Focus of fever**					*P* < 0.001
Upper respiratory tract infection	16,800	6,984	6,685 (95.7)	208 (3.0)	
Lower respiratory tract infection	4,456	2,763	2,663 (96.4)	85 (3.1)	
Gastro-intestinal illness	3,227	2,060	1,966 (95.4)	56 (2.7)	
Urinary tract	1,137	856	808 (94.4)	35 (4.1)	
Childhood exanthems/flulike illness	1,566	764	718 (94.0)	32 (4.2)	
Soft tissue/musculoskeletal	782	442	417 (94.3)	16 (3.6)	
Sepsis/meningitis	215	209	175 (83.7)	26 (12.1)	
Undifferentiated fever	2,417	1,208	1,061 (87.8)	124 (5.2)	
Inflammatory illness	123	68	53 (77.9)	11 (8.9)	
Other	883	456	412 (90.4)	26 (2.9)	
**Final diagnosis**					*P* < 0.001
IBI	57	55	48 (87.3)	6 (10.9)	
SBI	1,343	958	876 (91.4)	64 (6.7)	
Other	30,212	14,799	14,035 (94.8)	550 (3.7)	
**Disposition**					*P* < 0.048
Discharge	23,494	8,867	8,424 (95.0)	372 (4.2)	
Admission to general ward	8,003	6,773	6,441 (95.1)	240 (3.5)	
PICU admission	114	102	94 (92.2)	8 (7.8)	

Percentages are based on number of patients where lab tests were performed

PCT was most often performed in children below 3 months (12.0% versus 3.6% in older children, *p* < 0.001). PCT was used slightly more often in children with fever less than 24 h (4.9%) in comparison to children with a duration of fever ≥ 24 h (3.4%, *p* < 0.001). Regarding clinical alarming signs, PCT was used most often in children with meningeal signs (7.0%, range 0.0–66.7%) or a non-blanching rash (10.9%, range 0.0–94.9%).

Table 1 shows differences in CRP and PCT use in children with different clinical alarming signs, abnormal vital signs, disposition and focus of infection.

### Range between hospitals

We found large differences in CRP and PCT use between hospitals (appendix 2). CRP use ranged between 7.7–92% while PCT use ranged between 0.03% and 15.4%. When blood samples were taken, CRP use ranged between 79.8 and 99.9% and PCT use ranged between 0.1–85.5%.

In children below 3 months, the range of PCT use between hospitals was 0.0–93.3 and 0.0–84.8 in older children. Ranges of PCT use in children with fever less than 24 h and longer than 24 h were similar: 0–85%. PCT use in children with a clinical alarming sign present such as meningeal signs, a non-blanching rash or reduced consciousness ranged between 0.0 and 92%.

## Discussion

### Main findings

In our study of European EDs, PCT was scarcely used when evaluating febrile children. In three hospitals, PCT was not available at all. Large differences were found between the participating EDs in the use of PCT in general and for specific ages and clinical subgroups. PCT was used slightly more often in children were there is evidence that PCT offers improved sensitivity and specificity in comparison to CRP, [4, 9] such as short duration of fever or young infants, but overall, PCT use was low even in these subgroups [4].

### Findings in relation to previous literature

To our knowledge, only two previous studies on actual PCT use in non-neonatal febrile children were performed; both took place in the United States and only one took place at the ED [6, 10].

Dorney et al. showed similar low use of PCT (1.8%) in children attending the ED. As in our study, PCT use was highly variable between hospitals [6].

In a study by Cotter et al. among hospitalized children, PCT use was higher (12%) than in hospitalized children in our study [10]. Although PCT use was still lower than CRP (26% to 33%, depending on the diagnosis), differences between PCT and CRP use were less pronounced [10].

This low use of PCT may reflect the lack of clear guidance in the main national guidelines for febrile children. In our study, we did not assess what was recommended in each participating hospital's guideline on PCT use in febrile children. However, our previous study showed that the majority of hospitals used a guideline for febrile children based on the NICE guideline, which does not recommend PCT use [7, 11]. This statement was based on a literature search performed up to 2007. The most recent update of the NICE fever guideline in 2021 maintains this decision without discussing more recent studies, including several systematic reviews. Similarly, the Dutch national guideline on the evaluation and management of febrile children that was in place during the study period mentions that PCT is superior to CRP in evaluating young febrile children with a short duration of fever, however the general conclusion is that CRP and PCT have similar performance [12]. In this guideline, no concrete advice is given on the use of PCT in febrile children at the ED (e.g., in which children it should be used).

In contrast, in 2007, the American Academy of Pediatrics (AAP) published a clinical practice guideline on the evaluation and management of well-appearing febrile infants 8 to 60 days old where it is stated that for this pediatric age group, “procalcitonin has emerged as the most accurate inflammatory marker for risk stratification available.” and is recommended as a first-choice biomarker in all age groups [13].

Several studies have demonstrated the excellent diagnostic properties of PCT in children with IBI (sepsis or meningitis) [2, 4], children with a short duration of fever [4] and its cost-effectiveness in specific populations, such as children with meningococcal disease [14] or young febrile infants [9]. For example, the cost-effectiveness studies on febrile infants showed a potential cost reduction of 6–15% regarding hospital-level-door-to-discharge costs, including hospitalized infants with SBIs, hospitalized infants without SBIs and discharged infants subsequently hospitalized for missed SBIs [9].

In a meta-analysis on prodromal meningococcal disease [14], Bell et al. assessed the costs of four diagnosis groups (true positive, false negative, true negative and false positive) from a National Health Service payer perspective. Decision analytic model outcomes indicated that the incremental cost effectiveness ratio was £−8,137.25 (US $ −13,371.94) per correctly treated patient [14].

Similar cost-effectiveness studies in a broader ED population, e.g., children that attend the ED with undifferentiated fever, still have to be performed.

PCT performs similarly to CRP when outcome measures include both IBI and non-invasive SBI [3, 4]. Routine PCT testing in all febrile children is therefore not warranted. Updating guidelines to reflect evidence on PCT in specific pediatric populations—such as young febrile children, those at risk for invasive bacterial infections, and those with a short duration of fever—could improve evidence-based PCT use while acknowledging its limited value in all febrile children [9, 14]. As an example, the updated national Dutch guideline on febrile children, advices to perform PCT in the following situations: if fever has been present for less than 8–12 h; in case of a discrepancy between a strong clinical suspicion and a normal or marginally elevated CRP value or to assess the risk of an IBI as part of a SBI [15].

A more general explanation of the limited use of PCT is the phenomenon that – in general – there is a considerable time lag between the dissemination of study results and their application in practice, especially when these are not incorporated into guidelines. Morris et al. have shown that the average time to implement results is 17 years [16]. In a qualitative study, performed 18 months after the introduction of PCT in the participating hospital, physicians still reported a knowledge gap, which made them uncertain about appropriate procalcitonin use and interpretation [17].

A second important barrier is the lack of availability of PCT and its costs. For example, in some settings PCT is not available at the ED, cannot be performed from a capillary blood sample, or has to be sent to an external laboratory; the delays are then such that it is less pertinent to be used at the ED. Other barriers might include lack of physician knowledge on the available evidence and lack of readiness to adapt new clinical strategies. A BMJ study on evidence-based medicine [18] use by health care professionals, showed that only 14% of respondents used EBM regularly. When EBM sources were used, these were mostly guidelines; PubMed was used by less than half of the respondents and the Cochrane library by less than 10% of all respondents [18]. However, guidelines are not always updated as often as recommended. Furthermore, in a systematic review on the implementation of healthcare innovation, 15% of the studies reported that lack of readiness for change by the organization was reported to be an important barrier in the implementation of an innovation such as a new technique or method [19].

Waterfield et al. conducted an insightful study demonstrating that clinical guidelines are highly sensitive in identifying children with meningococcal disease, achieving a 100% detection rate with no missed cases. However, these guidelines were notably non-specific (a specificity of 0.0%), resulting in many unnecessary admissions and treatments. In contrast, clinician judgment reduced overtreatment but missed 11% of cases of meningococcal sepsis. This highlights the limitations of both current guidelines and clinician practice, emphasizing the need for additional tools with improved sensitivity as well as specificity, to enhance clinical decision-making [20].

Several clinical studies, using PCT either alone or as part of an algorithm, showed PCT to offer improved sensitivity and specificity and a very low rate of misclassification in comparison to traditional biomarkers or algorithms not using PCT [1, 2, 4, 9, 21–23].

### Implications for clinical practice and research

In the era of evidence-based medicine, further description of the evidence for the value of PCT testing in the febrile pediatric emergency population may contribute to its use in clinical practice Although several systematic reviews have been performed on the use of PCT in febrile children, they either did not separately analyze SBI (e.g. pneumonia, urinary tract infection)) and IBI (e.g. sepsis, meningitis) [1] or did not compare PCT to CRP directly [2]. Furthermore, to our knowledge, cost-effectiveness of PCT has only been studied in specific pediatric populations [14] and a cost-effectiveness study in a broader ED population still has to be performed.

The majority of biomarker studies focus on their development and validation in clinical practice. However, the implementation of these biomarkers in healthcare settings should also be investigated. A future study could focus on specific barriers to PCT use in the ED [19, 24].

### Strengths and limitations

The main strength of this study is the large number of study sites in different European countries and the large number of patients, adding to the external validity of our results. As we found considerable differences in PCT use between hospitals, potential differences in patient populations should be taken into account when interpreting studies on PCT use and when implementing new guidelines.

## Conclusion

Actual PCT use in febrile children at European EDs is limited and varies largely between hospitals. Possible explanations include lack of guidelines, limited availability, higher costs, a limited number of cost-effectiveness studies and a general time lag regarding the implementation of evidence into clinical practice.

## Supplementary Information

Supplementary Material 1.

Supplementary Material 2.

Supplementary Material 3.

## Data Availability

Individual participant data that underlie the results reported in this article, including a data dictionary, will be made available after de-identification to researchers who provide a methodologically sound proposal. Proposals should be directed to dm.borensztajn@nwz.nl. To gain access, data requestors will need to sign a data access agreement.

## Abbreviations

CRP: C-reactive protein
ED: Emergency Department
IBI: Invasive bacterial infections
MOFICHE: Management and Outcome of Fever in children in Europe
PERFORM: Personalized Risk assessment in Febrile illness to Optimize Real-life Management across the European Union
PCT: Procalcitonin
SBI: Serious bacterial infections
