# Supplementary material for: Procalcitonin use in febrile children attending European emergency departments: a prospective multicenter study
Source: BMC Pediatr. 2025 Mar 1;25:157. doi: 10.1186/s12887-025-05483-1 (PMC11871781; doi:10.1186/s12887-025-05483-1)
Supplement: Supplementary file 1 — Supplementary Material 1. [file 12887_2025_5483_MOESM1_ESM.docx]

**Appendix 1. CRP and PCT use in different age groups.**

|  | **Number of episodes** | **Lab tests performed** | **CRP without PCT (%)** | **PCT with or without CRP (%)** | **P-value** |
| --- | --- | --- | --- | --- | --- |
| **All children with fever:**  **N = 31,612** | 31,612 | 15,812 | 14,959 (94.6) | 620 (3.9) |  |
| **Age** |  |  |  |  | P< 0.001 |
| <3 months | 777 | 559 | 480 (85.9) | 67 (12.0) |  |
| 3-12 months | 4,609 | 2,254 | 2,134 (94.7) | 87 (3.9) |  |
| 1-4 years | 16,989 | 8,244 | 7,865 (95.4) | 297 (3.6) |  |
| 5-12 years | 7,062 | 3,409 | 3,202 (94.0) | 130 (3.8) |  |
| >12 years | 2,175 | 1,346 | 1,278 (94.9) | 39 (2.9) |  |
