# Supplementary material for: Procalcitonin use in febrile children attending European emergency departments: a prospective multicenter study
Source: BMC Pediatr. 2025 Mar 1;25:157. doi: 10.1186/s12887-025-05483-1 (PMC11871781; doi:10.1186/s12887-025-05483-1)
Supplement: Supplementary file 2 — Supplementary Material 2. [file 12887_2025_5483_MOESM2_ESM.docx]

**Appendix 2. PCT use in specific patient groups per centre (selection of children where laboratory tests were performed).**

|  | **Number of episodes** | **PCT with or without CRP (%)** |  |  |
| --- | --- | --- | --- | --- |
| **All episodes** | **15,812** | **620 (3.9)** |  |  |
| Liverpool, UK | 515 | 1 (0.2) |  |  |
| Newcastle, UK | 558 | 2 (0.4) |  |  |
| Rotterdam, the Netherlands | 1,029 | 20 (1.9) |  |  |
| Athens, Greece | 1,552 | 13 (0.8) |  |  |
| Santiago de Compostella, Spain | 372 | 318 (85.5) |  |  |
| Graz, Austria | 2,059 | 7 (0.3) |  |  |
| Riga, Latvia | 5,854 | 3 (0.1) |  |  |
| München, Germany | 479 | 181 (37.8) |  |  |
| Ljubljana, Slovenia | 3,394 | 75 (2.2) |  |  |
| **Age** | **< 3 months** |  | **> 3 months** |  |
|  | **Number of episodes** | **PCT with or without CRP (%)** | **Number of episodes** | **PCT with or without CRP (%)** |
|  | **559** | **67 (12.0)** | **15,253** | **553 (3.6)** |
| Liverpool, UK | 60 | 1 (1.7) | 455 | 0 (0.0) |
| Newcastle, UK | 73 | 2 (2.7) | 485 | 0 (0.0) |
| Rotterdam, the Netherlands | 42 | 3 (7.1) | 987 | 17 (1.7) |
| Athens, Greece | 77 | 0 (0.0) | 1475 | 13 (0.9) |
| Santiago de Compostella, Spain | 30 | 28 (93.3) | 342 | 290 (84.8) |
| Graz, Austria | 25 | 2 (8.0) | 2034 | 5 (0.2) |
| Riga, Latvia | 133 | 0 (0.0) | 5721 | 3 (0.1) |
| München, Germany | 30 | 14 (46.7) | 449 | 167 (37.2) |
| Ljubljana, Slovenia | 89 | 17 (19.1) | 3305 | 58 (1.8) |
| **Duration of fever** | **<24 hours** |  | **>24 hours** |  |
|  | **Number of episodes** | **PCT with or without CRP (%)** | **Number of episodes** | **PCT with or without CRP (%)** |
|  | **4,780** | **236 (4.9)** | **10,230** | **347 (3.4)** |
| Liverpool, UK | 175 | 0 (0.0) | 184 | 1 (0.5) |
| Newcastle, UK | 171 | 2 (1.2) | 263 | 0 (0.0) |
| Rotterdam, the Netherlands | 402 | 12 (3.0) | 505 | 6 (1.2) |
| Athens, Greece | 384 | 3 (0.8) | 1,141 | 10 (0.9) |
| Santiago de Compostella, Spain | 131 | 112 (85.5) | 214 | 182 (85.0) |
| Graz, Austria | 741 | 2 (0.3) | 1,152 | 4 (0.3) |
| Riga, Latvia | 1,548 | 0 (0.0) | 4,182 | 3 (0.1) |
| München, Germany | 130 | 57 (43.8) | 324 | 114 (35.2) |
| Ljubljana, Slovenia | 1,098 | 48 (4.4) | 2,265 | 27 (1.2) |
| **Any clinical alarming sign*** | **5,143** | **181 (3.5)** |  |  |
| Liverpool, UK | 217 | 1 (0.5) |  |  |
| Newcastle, UK | 308 | 2 (0.6) |  |  |
| Rotterdam, the Netherlands | 384 | 12 (3.1) |  |  |
| Athens, Greece | 68 | 0 (0.0) |  |  |
| Santiago de Compostella, Spain | 87 | 80 (92.0) |  |  |
| Graz, Austria | 363 | 6 (1.7) |  |  |
| Riga, Latvia | 2,553 | 0 (0.0) |  |  |
| München, Germany | 122 | 43 (35.2) |  |  |
| Ljubljana, Slovenia | 1,041 | 37 (3.6) |  |  |
| **Focus of fever:** |  |  |  |  |
|  |  |  |  |  |
| **Focus of fever:** | **Sepsis/meningitis** |  | **Undifferentiated fever** |  |
|  | **Number of episodes** | **PCT with or without CRP (%)** | **Number of episodes** | **PCT with or without CRP (%)** |
|  | **209** | **26 (12.4)** | **1,208** | **124 (10.3)** |
| Liverpool, UK | 29 | 0 (0.0) | 72 | 1 (1.4) |
| Newcastle, UK | 44 | 2 (4.5) | 54 | 0 (0.0) |
| Rotterdam, the Netherlands | 47 | 4 (8.5) | 129 | 6 (4.7) |
| Athens, Greece | 5 | 0 (0.0) | 33 | 0 (0.0) |
| Santiago de Compostella, Spain | 4 | 4 (100.0) | 95 | 86 (90.5) |
| Graz, Austria | 1 | 1 (100.0) | 98 | 1 (1.0) |
| Riga, Latvia | 19 | 0 (0.0) | 418 | 0 (0.0) |
| München, Germany | 14 | 8 (57.1) | 33 | 13 (39.4) |
| Ljubljana, Slovenia | 46 | 7 (15.2) | 276 | 17 (6.2) |

Percentages are based on number of patients in the first column.
* Clinical alarming signs: ill appearance, non-blanching rash, abnormal consciousness, meningeal signs or neurological signs.
